# Pathological cytomorphologic features and the percentage of ALK FISH-positive cells predict pulmonary adenocarcinoma prognosis: a prospective cohort study

**DOI:** 10.1186/s12957-021-02386-0

**Published:** 2021-09-16

**Authors:** Fenge Jiang, Congcong Wang, Ping Yang, Ping Sun, Jiannan Liu

**Affiliations:** 1 Department of Oncology, The Affiliated Yantai Yuhuangding Hospital of Qingdao University, Yantai, Shandong 264000 People’s Republic of China; 2 Department of Pathology, The Affiliated Yantai Yuhuangding Hospital of Qingdao University, Yantai, Shandong 264000 People’s Republic of China

**Keywords:** Pathological cytomorphologic, ALK FISH positive, Prognosis, Crizotinib treatment

## Abstract

**Background:**

We conducted a study to explore the relationship between pathological cytomorphologic features and the percentage of anaplastic lymphoma kinase (ALK)-positive cells to better predict pulmonary adenocarcinoma prognosis with crizotinib treatment.

**Patients and methods:**

We investigated 60 cases of patients with ALK-positive advanced or metastatic non-small cell lung cancer (NSCLC). Immunohistochemistry was performed to screen for ALK rearrangement. Fluorescence in situ hybridization (FISH) was used to detect the percentage of ALK-positive cells. The primary objectives of the study were the progression-free survival (PFS), the 3-year overall survival, and the 3-year overall survival (OS) rates. The secondary objectives of the study were the disease control rate (DCR) and the overall response rate (ORR).

**Results:**

We compared the pathological cytomorphologic features of 60 cases of ALK-positive pulmonary adenocarcinoma, of which 21 cases were ALK-positive with signet ring cell cytomorphologic characteristics. There were statistical differences in the ORR (*p* = 0.019), DCR (*p* = 0.032), and PFS (*p* = 0.047) between the signet ring cell group and group without signet ring cells. Of these, 37 cases were ALK-positive with EML4 (echinoderm microtubule associated protein like 4)-ALK high percentage of positivity group. These cases benefited more from crizotinib treatment in the ORR (*p* = 0.046) and achieved a longer PFS (*p* = 0.036) compared to those with EML4-ALK low percentage of positivity group.

**Conclusions:**

Signet ring cell cytomorphologic characteristics of pulmonary adenocarcinoma are associated with the percentage of ALK-positive cells. Signet ring cell cytomorphologic characteristics and the percentage of ALK-positive cells might predict the prognosis of pulmonary adenocarcinoma with crizotinib treatment.

**Trial registration:**

The study was approved by the Institutional Review Board (Medical Ethics Committee of Yantai Yuhuangding Hospital). The registration number is NO.2016[193].

## Introduction

Pulmonary adenocarcinoma is the most common histological type of primary lung cancer and is a heterogeneous tumor with diverse clinical and molecular pathological characteristics [1–3]. The 2015 World Health Organization (WHO) classification of pulmonary adenocarcinoma was published with numerous significant changes from the 2004 WHO classification [4, 5]. The new classification abolishes the subtypes of clear cells and signet ring cells (SRCs) and determines these as cytomorphologic characteristics. SRCs are characterized by singly dispersed tumor cells with intracytoplasmic mucin and an eccentrically displaced nucleus. They are a rare feature of primary pulmonary adenocarcinoma [6]. However, this feature should not be neglected no matter how few cells are present because its existence has discriminative significance for pulmonary adenocarcinoma. Many studies have reported that SRCs are the most salient feature of anaplastic lymphoma kinase (ALK)-positive pulmonary adenocarcinoma [7]. This characteristic could serve as the most vital criterion for the identification of cases that have a probability of harboring ALK rearrangement. However, no research about the correlations between this cytomorphological feature and the prognosis of crizotinib treatment in ALK-positive pulmonary adenocarcinoma has been conducted.

As a receptor tyrosine kinase, ALK belongs to the insulin receptor tyrosine kinase family [8, 9]. The ALK gene has been found to be rearranged, mutated, or amplified in a series of tumors, including anaplastic large cell lymphoma, neuroblastoma, and non-small-cell lung cancer (NSCLC) [10]. With the development of tumor molecular targeted therapy, ALK now plays an important role in the oncogenesis of NSCLC [11]. Rearrangements of ALK are present in 3–7% of NSCLC cases, with echinoderm microtubule associated protein like 4 (EML4)-ALK translocation being the most common; other fusion partners, such as kinesin family member 5B (KIF5B), kinesin-light chain 1 (KLC1), and translocated promoter region (TPR), occur only in 5% [12]. Despite ALK-positive patients accounting for a small proportion of NSCLC, the absolute number of patients with ALK-positive NSCLC is not small due to the greater worldwide incidence of lung cancer [13]. Different methods for detecting patients with ALK-positive NSCLC are now available, such as immunohistochemistry (IHC), fluorescence in situ hybridization (FISH), and next-generation sequencing [14–16]. Of these, FISH is considered to be a “gold standard” to guide ALK inhibitor treatment in clinical settings [17]. Although FISH cannot detect different ALK genetic fusion partners or multiple distinct EML4-ALK chimeric variants, it can distinguish the percentage of ALK-positive cells [4, 18]. The ALK gene rearrangements occur between a 5′ fusion partner and its promoter encoding the 3′ ALK kinase domain. An ALK break-apart DNA probe labels the telomeric 3′ part of the fusion breakpoint in orange and the centromeric 5′ part in green [6]. A case is considered positive for ALK rearrangement if > 15% of cells showed split signals [7]. Many studies have only focused on the higher percentages of ALK-positive cells associated with larger differences in the overall response rate (ORR) and progression-free survival (PFS) between ALK tyrosine kinase inhibitor (TKI) and chemotherapy. No research on the association between the extent of ALK FISH positivity and the outcomes of ALK-TKI has been carried out.

Now, ALK inhibitor crizotinib is the standard first-line treatment for ALK-positive locally advanced and metastatic NSCLC [19]. Crizotinib has demonstrated superior response rates and PFS compared with chemotherapy [8, 20] and the median overall survival (OS) of ALK-positive NSCLC cases treated with crizotinib may be more than 5 years [21]. Newer-generation ALK-TKIs, including ceritinib, alectinib, brigatinib, have demonstrated efficacy for ALK-positive pulmonary adenocarcinoma [22–24]. However, not everyone benefits from crizotinib, which might be due to tumor heterogeneity, different tumor staging, different multidrug resistance mechanisms, and so on. In our research, we focus on the cytomorphologic characteristics, percentage of ALK positivity, and relationship with crizotinib treatment of pulmonary adenocarcinoma.

## Materials and methods

### Patient characteristics

This was a single-center, single-arm study from the Yuhuangding Hospital affiliated with Qingdao University between August 2017 and January 2019.

The inclusion criteria includes: (i) age ≥ 18 years and ALK-positive pulmonary adenocarcinoma confirmed by IHC and FISH. All tumors of these patients were evaluated by at least one measurable lesion through computed tomography scanning. (ii) Eastern Cooperative Oncology Group (ECOG) score of 0~2. (iii) Patients treated with crizotinib as a first-line therapy. (iv) Life expectancy of more than 3 months. (v) Informed consent obtained from all patients before treatment. The exclusion criteria includes: (i) Normal routine blood tests with liver and kidney functions > 1.5 times the normal range. (ii) Patients had received other anti-tumor treatments including chemotherapy and radiotherapy before being treated with crizotinib.

The study was approved by the Institutional Review Board (Medical Ethics Committee of Yantai Yuhuangding Hospital).

### Methods

All patients were treated with crizotinib at 250 mg twice daily.

### Efficacy assessment

(i) Short-term effects: The objective efficacy was evaluated by RECIST 1.1 [25]. Complete response (CR) was defined as the disappearance of all tumors after crizotinib treatment. Partial response (PR) was defined as a decline of 30% at least in the maximum tumor diameters, and progressive disease (PD) was defined as an increase of 20% at least from the baseline in the sum measure of all tumor diameters. The disease was categorized as stable disease (SD) when CR, PR, or PD was not noted. The ORR was defined as patients showing CR and PR. The disease control rate (DCR) was defined as patients showing CR, PR, and SD.

(ii) Long-term effects: Here, PFS was defined as the time from the first use of crizotinib to the progression of disease. The OS was defined as the time from the date of biopsy to death or the last follow-up.

### Pathological cytomorphologic assessment

Tumor biopsy samples were taken from the primary or metastatic site through transthoracic and endobronchial biopsy. All biopsied specimens were fixed with formalin and stained with hematoxylin and eosin. Two independent pathologists examined an average of 5.6 slides (range from 2 to 8) for every specimen. We not only identified the major subtypes, such as solid, papillary, acinar, and lepidic, but also recorded other cell morphological features, including the presence of SRCs, extracellular mucin, and cribriform growth pattern. All cytomorphological features in all present growth components were evaluated in three fields of each growth pattern using high magnification (× 400).

### IHC and scoring for ALK

A Ventana ALK assay using D5F3 antibody (Ventana Medical Systems, Tucson, AZ, U.S.A.) was performed to detect ALK rearrangement [26]. The IHC results were defined as 0 (none), 1+ (faint cytoplasmic staining, ≥ 10% of tumor cells), 2+ (moderate, smooth cytoplasmic staining), and 3+ (intense, granular cytoplasmic staining). Here, IHC scores of 2+ or 3+ were regarded as ALK-positive.

### ALK FISH analysis

The ALK FISH analysis was performed with the Vysis ALK Break Apart FISH Probe Kit (Abbott Molecular, Des Plaines, IL, U.S.A.) according to the manufacturer’s instructions [27]. To start, 50 cells were counted, and a sample was considered as negative if < 5 cells were FISH-positive and positive if > 25 cells were FISH-positive. The results were equivocal if 5 to 25 cells had a positive pattern. An additional 50 cells were enumerated, and the sample was considered as positive if the average percent of the positive cells for the two scoring assessments was ≥ 15%. For the purpose of this study, the cut-off value differentiating high and low ALK positivity was ≥ 50%.

### Follow-up

The survival data were collected by telephone interviews and outpatient service. The follow-up time was from the time the patients enrolled in the study to February 1, 2021.

### Statistical analysis

We used the SPSS 20.0 software for the statistical analysis. Descriptive statistical methods were used for the baseline characteristics. Dichotomous variables were expressed in terms of the number of patients and percentages, and continuous variables were expressed in terms of median and range values. The survival curves were constructed by the Kaplan–Meier method, and the differences were analyzed by the log-rank test. All statistical tests were two-tailed, with *p* < 0.05 considered statistically significant.

## Results

### The clinical data of the enrolled patients

According to the entry criteria, 60 patients with ALK-positive pulmonary adenocarcinoma were enrolled in this research at the Oncology Department of Yuhuangding Hospital, Yantai, China. The median age was 55 (ranging from 27 to 80), and 36 (60.0%) were female and 24 (40.0%) male. Moreover, 49 (81.7%) were non-smokers, and 11 (18.3%) were smokers; 23 (38.3%) were stage IIIA–IIIB, and 37 (61.7%) were stage IV. Finally, 55 (91.7%) had a good ECOG PS of 0-1. All resected tumors were classified according to the histologic subtype based on the new International Association for the Study of Lung Cancer/American Thoracic Society/European Respiratory Society classification [28]; the predominant pattern was determined as acinar predominant in 26 cases, solid predominant in 13 cases, papillary predominant in 10 cases, micropapillary predominant in 4 cases, and lepidic predominant in 7 cases. All other components (> 5% of tumors) were also noted, including the presence of SRCs, extracellular mucin, and cribriform growth pattern. The baseline patient characteristics are shown in Table 1.

**Table 1:** Clinicopathologic features of ALK+ lung adenocarcinoma

Characteristic	No. of patients (%)
Age, years	
Median	55
Range	27–80
< 60	39 (65.0%)
≥ 60	21 (35.0%)
Gender	
Male	24 (40.0%)
Female	36 (60.0%)
Smoking status	
Non-smoker	49 (81.7%)
Smoker	11 (18.3%)
Clinical staging	
IIIA-IIIB	21 (35.0%)
IV	39 (65.0%)
ECOG PS	
0–1	55 (91.7%)
2	5 (8.33%)
Predominant pattern	
Acinar	26 (43.3%)
Solid	13 (21.7%)
Papillary	10 (16.7%)
Micropapillary	4 (6.7%)
Lepidic	7 (11.7%)
Microscopic cytomorphologic findings	
Signet ring cells	21 (35.0%)
Extracellular mucin	19 (31.7%)
Cribriform pattern	12 (20.0%)

### Correlation of pathological cytomorphologic characteristics and the response to crizotinib in patients with ALK-positive NSCLC

**Fig. 1:**
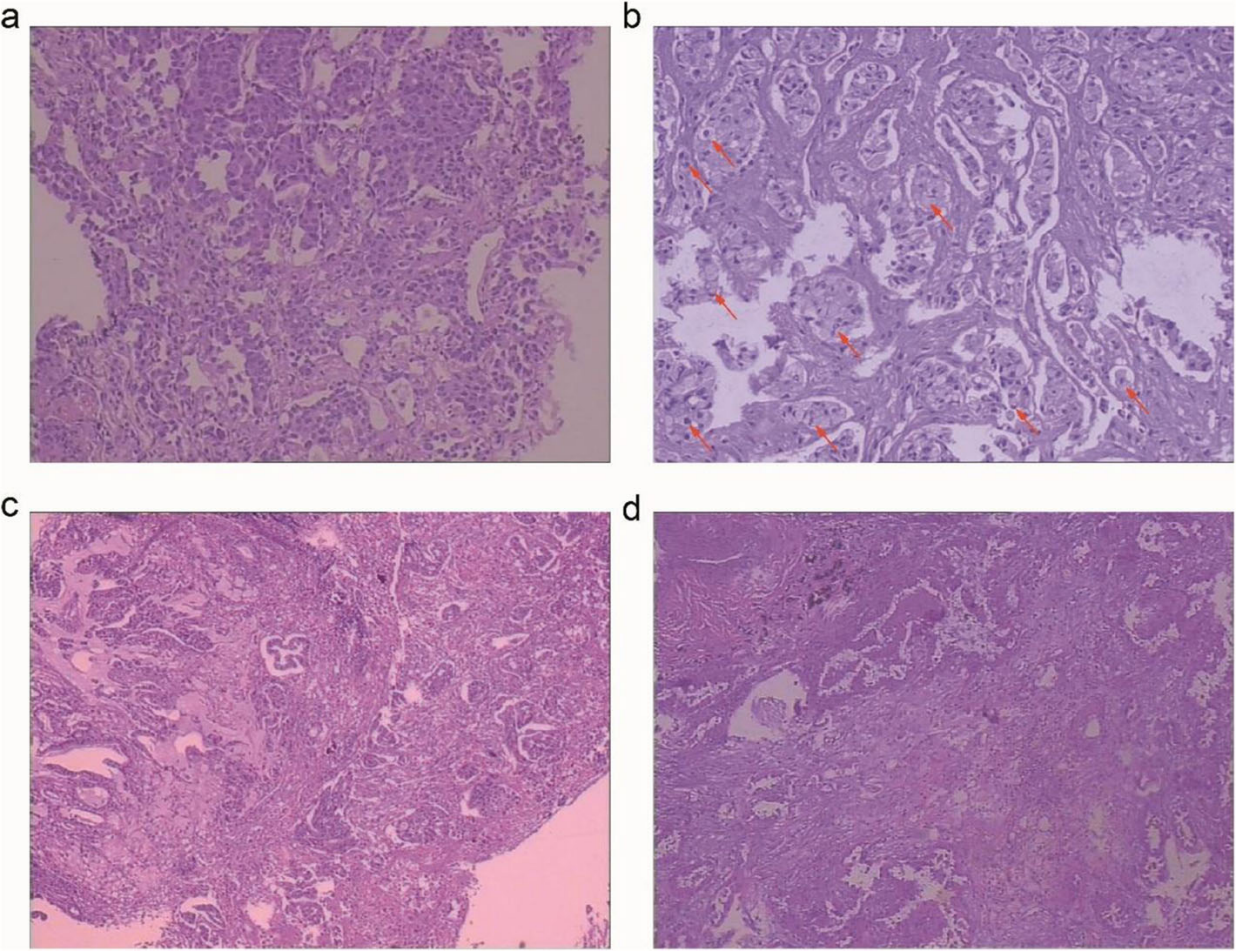
Cytomorphologic of lung adenocarcinomas harboring ALK rearrangements. **a** ALK-rearranged lung adenocarcinoma (× 200). **b** ALK-rearranged lung adenocarcinoma with signet ring cells (red arrows indicate signet ring cells) (× 400). **c** *ALK*-rearranged lung adenocarcinoma with extracellular mucin (× 100). **d** ALK-rearranged lung adenocarcinoma with cribriform pattern (× 100)

In our study, 43.3% of cases showed acinar-predominant growth patterns, and 21.7% of cases showed solid-predominant growth patterns in the patients with ALK-positive lung cancer (Table 1). We focused on the association between cytomorphologic features, such as SRC components, extracellular mucin, cribriform pattern, and ALK- positive lung cancer (Fig. 1). SRC carcinoma was the most common pattern (35.0%), followed by the extracellular mucin (16.7%) (Table 1).

The ORR and the DCR responded to crizotinib in 60 cases of ALK-positive NSCLC at 75.0% (45/60) and 86.7% (52/60), respectively. Compared to the SRC carcinoma groups, there was a higher ORR (84.6% vs. 57.1%, *p* = 0.019, Table 2) and DCR (94.9% vs. 71.4%, *p* = 0.032, Table 2) in the groups without SRC. No difference was found between the other pathological cytomorphologic characteristics, ORR, or DCR.

**Table 2:** Correlation of cytomorphologic pathological character and the response to crizotinib in ALK-positive NSCLC patients

Cytomorphologic pathological features	ORR (%) *P* value		*P* value	DCR (%)
Signet ring cells		0.019		0.032
Yes (*N* = 21)	57.1% (12/21)		71.4% (15/21)	
No (*N* =39)	84.6% (33/39)		94.9% (37/39)	
Extracellular mucin		0.631		0.806
Yes (*N* = 19)	68.4% (13/19)		84.2% (16/19)	
No (*N* = 41)	78.0% (32/41)		90.2% (37/41)	
Cribriform pattern		0.264		0.393
Yes (*N* = 12)	58.3% (7/12)		75% (9/12)	
No (*N* = 48)	79.2% (38/48)		89.6% (43/48)	

**Fig. 2:**
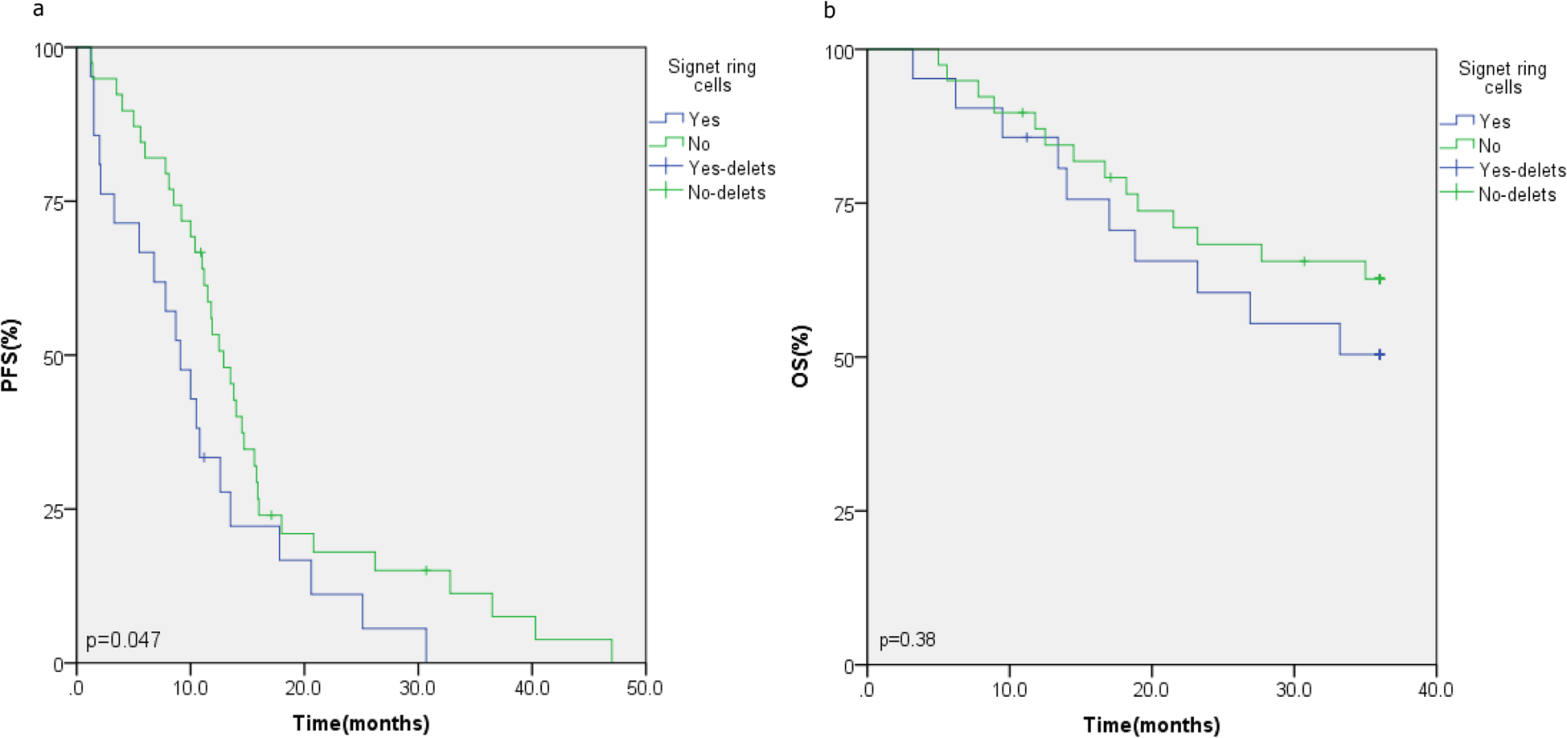
Comparison of PFS (**a**) and OS (**b**) in patients with signet ring cells vs without signet ring cells

Of the 60 cases, 56 cases were followed up with and four cases lost to follow-up by the end of February 1, 2021. The median follow-up time of the 56 cases was 42.5 months (ranging from 36.2 to 58.6 months). The median PFS was 11.5 months. The median OS was not reached. The median PFS (12.9 vs. 9.1 months, *p* = 0.047, Fig. 2a) was better in the group without SRCs compared to the group with SRCs. Although no difference in the 3-year OS (*p* = 0.38, Fig. 2b) was found between these two groups, the OS in the group without SRC had a longer trend than that in the SRC group, and the OS of the two groups did not reach the study endpoint. We analyzed the 3-year OS rates of the two groups, and the results showed that there was no statistical significance between the groups with SRC and without SRC (47.6% vs. 56.4%, *p* = 0.515).

### Correlation of the percentage of ALK positivity and the response to crizotinib in patients with ALK-positive NSCLC

**Fig. 3:**
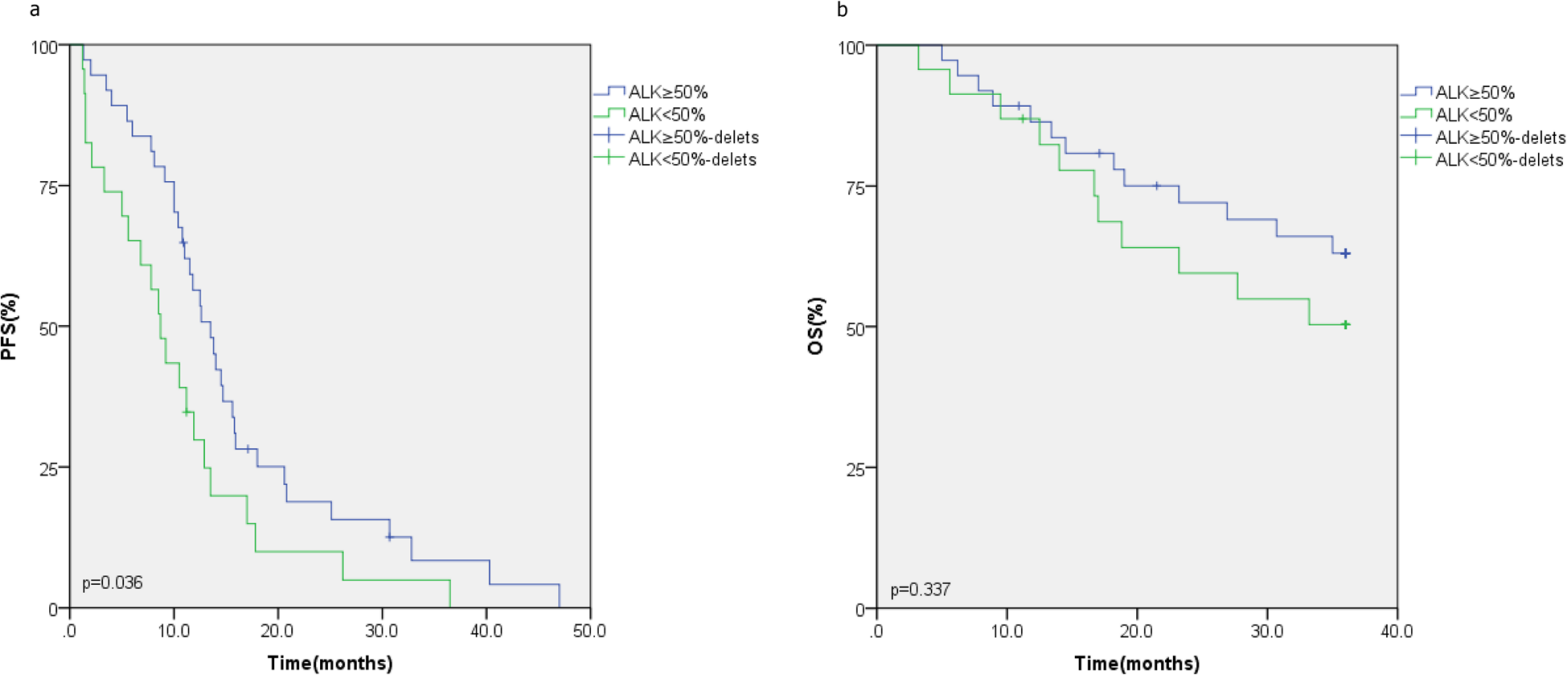
Comparison of PFS (**a**) and OS (**b**) in patients with ALK FISH ≥ 50% positive nuclei vs. < 50% positive nuclei

We defined the cut-off value as ≥ 50% to differentiate high and low ALK positivity. A total of 61.7% (37/60) of patients with ALK rearrangement had high positivity, whereas 38.3% (23/60) of patients had low positivity. We compared the response to crizotinib of the patients with high and low ALK-positive rearrangement. Overall, the patients with high ALK positivity responded better to crizotinib than the patients with low ALK positivity in the ORR (83.8% vs. 60.9%, *p* = 0.046, Table 3). There was no statistical significance in the DCR (89.2% vs. 82.3%, *p* = 0.735, Table 3). Compared to the low ALK positivity group, the PFS (13.5 vs. 8.7 months, *p* = 0.036, Fig. 3a) was longer in the high ALK positivity group. The 3-year OS (*p* = 0.337, Fig. 3b) of the two groups was not statistically significant. The OS of the two groups did not reach the study endpoint. We analyzed the 3-year OS rates of the two groups, and the results showed that there was no statistical significance between the high ALK positivity group and the low ALK positivity group (56.7% vs. 47.8%, *p* = 0.500).

**Table 3:** Correlation of the percentage of ALK-positive and the response to crizotinib in ALK-positive NSCLC patients

Response	NO. of patients (*N* = 60) (%)		*P* value
	ALK-positive ≥ 50% (*N* = 37)	ALK-positive < 50% (*N* = 23)	
ORR,%	83.8% (31/37)	60.9% (14/23)	0.046
DCR,%	89.2% (33/37)	82.3% (19/23)	0.735

### Correlation of the pathological cytomorphologic characteristics and the percentage of primary ALK+ lung adenocarcinoma

Approximately 56.5% (13/23) of the patients with the SRC pattern were identified in the low ALK positivity group, while only 21.6% (8/37) of the patients with the SRC pattern were found in the high ALK positivity group (*p* = 0.006, Table 4). No other cytomorphologic parameters were found to be associated with the percentage of ALK positivity.

**Table 4:** Correlation of the cytomorphologic pathological character and the percentage of primary ALK+ lung adenocarcinomas

Cytomorphologic pathological character	NO. of patients (*N* = 60)		*P* value
	ALK-positive ≥ 50% (*N* = 37)	ALK-positive < 50% (*N* = 23)	
Signet ring cells			0.006
Yes (*N* = 21)	8	13	
No (*N* = 39)	29	10	
Extracellular mucin			0.453
Yes (*N* = 19)	11	9	
No (*N* = 41)	26	14	
Cribriform pattern			0. 207
Yes (*N* = 12)	5	7	
No (*N* = 48)	32	16	

## Discussion

First identified in anaplastic large cell lymphomas, ALK is a receptor protein tyrosine kinase that activates many downstream signaling pathways, resulting in increased cell proliferation and survival [29]. Now, in many different cancers, such as neuroblastoma [30], and non-small cell lung cancer [31], rearrangements of ALK are found. This makes ALK an attractive target for cancer therapy. The ALK rearrangements are mostly ascribed to EML4-ALK fusion, and NSCLC harboring ALK rearrangements expresses a marked responsiveness to ALK inhibitors [32]. In 2011, crizotinib was first approved by the United States Food and Drug Administration to treat locally advanced or metastatic ALK-positive lung adenocarcinoma [33, 34]. Compared to standard platinum-doublet chemotherapy, the first-line treatment of crizotinib has a longer PFS and OS [35]. In 2013, crizotinib was approved by the China Food and Drug Administration [36]. With the development of immunotherapy for NSCLC, targeted ALK-TKI molecular therapy is still the first-line treatment for the ALK rearrangements of NSCLC. Although the newest edition of NCCN guidelines indicate that the newer-generation ALK inhibitors, alectinib preferred the recommendation to treat ALK-positive NSCLC. In China, crizotinib may be cost-effective compared to standard chemotherapy and other ALK inhibitors in the Chinese healthcare system [37].

Although crizotinib has been proclaimed to be a breakthrough, a significant proportion of ALK-positive patients do not show clinical benefits. In our study, we collected 60 ALK-positive cases with IHC and FISH in order to exclude false positives or false negatives. The clinicopathology characteristics of ALK-positive NSCLC in our study were consistent with those of previous studies [38–41]. The differences in our study were focused on the presence of SRC components, cribriform pattern, extracellular mucin, and so on. SRCs are more common in stomach, colon, and appendiceal adenocarcinoma [42]. Primary SRC carcinoma of the lungs is a very rare disease [6, 43]. Several studies have confirmed that SRCs are the most significant independent feature of ALK-positive pulmonary adenocarcinoma [44–46]. The new 2015 WHO classification has abolished the subtypes of SRC and determined them to be cytomorphologic characteristic s[5]. However, studies have reported that at least 10% of ALK-positive patients in North America are associated with SRCs [47, 48]. In our study, we found SRCs in 21.6% of the patients with ALK-positive NSCLC. We further analyzed the cytomorphologic characteristic and its association with the prognosis of crizotinib treatment. The results showed that the patients with SRCs had a worse ORR (*p* = 0.019) and DCR (*p* = 0.032) and shorter PFS (*p* = 0.047). Although the 3-year OS (*p* = 0.38) did not reach statistical significance, it showed a decrease trend in the patients with SRCs. This might be related to the higher malignancy and poorer prognosis of SRC carcinoma. However, it did not exclude the probability that patients with SRCs have a worse prognosis from crizotinib treatment. Combined treatment therapy might be applicable to patients with the pathological cytomorphologic characteristic of SRCs.

Different methods for detecting patients with ALK-positive NSCLC are now available; FISH is the most validated technique used in clinical diagnosis [49, 50]. In our study, we detected the percentage of ALK with FISH and defined 50% as the cut-off value. The results showed that the patients with higher ALK positivity had a better ORR (*p* = 0.046) and longer PFS (*p* = 0.036) than the patients with lower ALK positivity. This was concordant with a large meta-analysis performed by Soria et al. [51]. There was no statistical significance in the DCR (*p* = 0.735), OS (*p* = 0.337), or OS rate (*p* = 0.500), which may have been the result of the small sample size and short follow-up time. Many factors, such as false positivity by FISH, specimen type, and testing technology, could affect the response to crizotinib treatment. The 50% cut-off was empirically determined to differentiate high or low ALK positivity. The accuracy of the cut-off value needs to be further confirmed in a larger-sample study. However, the results told us the higher percentage of ALK positivity, the better the prognosis of crizotinib treatment at least. Furthermore, with widespread use of next-generation sequencing to detect rarer uncommon ALK fusion variants and mutations, more suitable patients who could benefit from crizotinib treatment will be screened [52].

Upon further study, we found that the SRC component was mainly present in the cases with low ALK positivity (*p* = 0.006). Although the detection of this cytomorphologic characteristic and the percentage of ALK positivity cannot determine all patients who will have a better prognosis from crizotinib treatment, it could be applied as an ancillary method to identify patients who will benefit more from crizotinib treatment.

In conclusion, our study showed that the SRC cytomorphologic characteristic is associated with the percentage of ALK positivity. The SRC cytomorphologic characteristic and the percentage of ALK positivity might predict the prognosis of pulmonary adenocarcinoma with crizotinib treatment. But our study also had several limitations: first, our sample size was small and all cases were from a single center; this might lead to selection bias. Furthermore, the 50% cut-off was empirically determined to differentiate high or low ALK positivity, but the accuracy of the cut-off value was not confirmed in a larger-sample study. Thus, further studies with larger sample size are needed to confirm this conclusion.

## Data Availability

All data generated or analyzed during this study are included in this published article.

## Abbreviation

WHO: World Health Organization
SRCs: Signet ring cells
ALK: Anaplastic lymphoma kinase
NSCLC: Non-small-cell lung cancer
EML4: Echinoderm microtubule associated protein like 4
KIF5B: Kinesin family member 5B
KLC1: Kinesin-light chain 1
TPR: Translocated promoter region
IHC: Immunohistochemistry
FISH: Fluorescence in situ hybridization
ORR: Overall response rate
PFS: Progression-free survival
TKI: Tyrosine kinase inhibitor
OS: Overall survival
ECOG: Eastern Cooperative Oncology Group
CR: Complete response
PR: Partial response
PD: Progressive disease
SD: Stable disease
DCR: Disease control rate
